# Demand Prediction and Optimal Allocation of Shared Bikes Around Urban Rail Transit Stations

**DOI:** 10.1007/s40864-022-00183-w

**Published:** 2022-12-13

**Authors:** Liang Yu, Tao Feng, Tie Li, Lei Cheng

**Affiliations:** 1 School of Civil Engineering, Beijing Jiaotong University, No.3 Shangyuancun, Haidian District, Beijing, 100044 People’s Republic of China; 2 Urban and Data Science, Graduate School of Advanced Science and Engineering, Hiroshima University, 1-5-1 Kagamiyama, Higashi-Hiroshima, Hiroshima, 739-8529 Japan; 3 Bejing Metro Network Administration Co.,Ltd, Beijing Jingtou Building, No.6 Xiaoying North Road, Chaoyang District, Beijing, People’s Republic of China

**Keywords:** Bike sharing, Demand prediction, Bike-sharing deployment, Hybrid model, Big data

## Abstract

The imbalance between the supply and demand of shared bikes is prominent in many urban rail transit stations, which urgently requires an efficient vehicle deployment strategy. In this paper, we propose an integrated model to optimize the deployment of shared bikes around urban rail transit stations, incorporating a seasonal autoregressive integrated moving average with long short-term memory (SARIMA-LSTM) hybrid model that is used to predict the heterogeneous demand for shared bikes in space and time. The shared bike deployment strategy was formulated based on the actual deployment process and under the principle of cost minimization involving labor and transportation. The model is applied using the big data of shared bikes in Xicheng District, Beijing. Results show that the SARIMA-LSTM hybrid model has great advantages in predicting the demand for shared bikes. The proposed allocation strategy provides a new way to solve the imbalance challenge between the supply and demand of shared bikes and contributes to the development of a sustainable transportation system.

## Introduction

Bike sharing has become a popular transport option in many cities because it is green and convenient and an essential feeder for other transportation modes such as buses and metro. In the emerging trend of mobility-as-a-service (MaaS) system deployment, which involves multimodal transportation, bike sharing may be promoted even further. As an important transport connector, shared bikes could increase the attractiveness of urban transit and thus potentially encourage transit use and contribute to sustainable transportation. Bicycle sharing complements and enhances public transportation [1]. As manifested in many cities, shared bikes are often used in combination with transit, shortening the travel time induced by the access/egress stages of a complete journey by transit. Especially for urban rail transportation, bike sharing has become the leading way to connect the last mile of rail transportation [2].

Shared bikes are traditionally dock-based because of the management and payment requirement and have also recently emerged as dockless. While the latter is considered more convenient because of the parking convenience, many problems remain, such as disordered parking, abandoned bikes, and illegal space occupation. Rail transit stations, as hot spots for shared bicycles, make this contradiction unprecedentedly acute. On the one hand, the excessive influx of shared bicycles is not conducive to the organization and evacuation of passengers. On the other hand, insufficient bicycle storage will lead to passengers being forced to choose other ways to complete their journeys, reducing passenger comfort and leading to a decrease in traffic accessibility [3]. Both stations and bike-sharing operators have made efforts to this end. For example, planners can enhance resilience of urban transport networks by fully considering the capacity and the usage of bike-sharing docks, as well as the coherence of metro stations and bike-sharing docks, in distributing and rebalancing activities [4]. Some scholars have also proposed the joint operation of bike sharing and metro mainly to serve rail stations, and the results show that the total social cost can be reduced to a great extent [5]. However, the imbalance issue between demand and supply in space and time underlying the use of shared bikes is still prominent in the sense that the use rate of shared bikes at a specific location could be highly time-dependent, e.g., commuters pick up bikes in the morning peak, and varied in the different built environment around the station, e.g., central business districts (CBD) and residential neighborhoods. Therefore, it remains critical for shared bike operators and urban management bureaus to better coordinate the deployment of shared bikes in different places and at different times of the day.

Many studies have addressed the issues related to shared bikes from different perspectives, which provides a theoretical basis for the better planning of shared bikes around urban rail transit stations. Five main aspects were discussed in the literature, namely, the factors affecting the use of shared bikes [6], the characteristics of shared bike users [7], the spatial and temporal distribution of bike use [8, 9], the demand prediction of shared bikes [10, 11], the allocation of shared bikes [12], and the integration of shared bikes with transit [13]. These studies offer valuable insights into shared bikes planning, but there are still various remaining problems, especially in the demand forecasting and allocation strategy.

The prediction of demand for shared bikes is essential for planning bike-sharing deployment, because the optimal allocation strategy can only be obtained when the predicted bike-sharing demand is close to the actual deployment needs. Existing research has developed different models by focusing on either the demand prediction or allocation strategy using actual data. There is a lack of integrated models which combine the demand prediction and bike allocation. We argue that the separate models may be more useful for a specific purpose. In contrast, models integrating the two are more useful to effectively realize the balance between supply and demand and have more practical significance.

In this study, therefore, we propose an improved bike-sharing allocation strategy to optimize the bike allocation around urban rail transit stations. A hybrid model based on time series and deep learning prediction theory is proposed, combining a seasonal autoregressive integrated moving average (SARIMA) model and a long short-term memory (LSTM) model to predict the usage of shared bikes around urban rail transit stations. Based on production and sales balance theory, a model to optimize bike allocation while minimizing the total dispatch cost is developed. Using 3-month big data of shared bikes in Beijing, the dynamic deployment strategy around urban rail transit stations is obtained by combining the actual dispatch process for shared bikes to meet the demand for shared bicycles during peak periods.

The remainder of this paper is organized as follows: Section 2 reviews the related literature on bike-sharing demand forecasting and deployment planning. Section 3 introduces the methodology employed in this study. Section 4 describes a case study of bike sharing in Beijing. The last section concludes this paper.

## Literature Review

The purpose of the literature review is twofold: to discuss the relevant work on demand prediction and the allocation of shared bikes. These two aspects of research have been primarily conducted separately in the existing literature. The purpose of this review is not to offer a systematic review of the research on shared bikes but rather to present the content-wise research and the methods related to the two perspectives.

The bike-sharing demand prediction has been discussed in different spatial scales, namely the city level, station clustering level, and single station level. City-level forecasting aims to predict coarser-grained bike usage at all stations throughout the city. Borgnat et al. [14], based on the data generated by the bike-sharing system in Leon City, developed a combination model to predict the hourly demand for shared bikes within a city. The results help provide an understanding of the travel characteristics of bike-sharing users. Based on the assumption that geographically adjacent stations have similar characteristics of time demand, Chen et al. [15] and Feng et al. [16] classified stations into different clusters and predicted station-based demand for the different categories. Lin et al. [17] and Wang et al. [18] showed that, although the prediction with station clustering can better capture the local demand than city-level results, the local differences between different bike-sharing stations are not addressed.

In the case of the station-based prediction models, Sohrabi et al. [19] proposed a two-step prediction method based on historical traffic and spatiotemporal characteristics to predict the demand at bike-sharing stations dynamically. Wu et al. [20] found that the current site-level bike-sharing demand prediction did not fully mine the information in the ordering data and ignored the potential relationship between different sites/stations. Machine learning models were used to predict the time-by-time demand for shared bikes in different sites.

Among the studies on demand prediction, time-series regression models have most typically been applied. Kaltenbrunner et al. [21] used the classic autoregressive moving average model (ARMA) to predict the number of shared bikes. Realizing that the ARMA model requires stationarity of the time series, Yoon et al. [22] used the autoregressive integrated moving average (ARIMA) model to predict the demand of each station for shared bikes, such that the stationarity problem of the time series in the number of shared bikes is solved by setting the time cycle. Apart from the statistical models, recent efforts have also involved the use of machine learning methods. For example, Liu et al. [23] developed a demand prediction model for shared bikes using an artificial neural network.

In addition, scholars have increasingly begun to combine different types of methods to predict the demand for shared bicycles. Mehdizadeh et al. [24] used a hybrid convolutional neural network (CNN)-LSTM model to predict bicycle demand during the COVID-19 pandemic, and the results showed higher accuracy than ARIMA. A model combining LSTM and gated recurrent units (GRU) was proposed by Boonjubut et al. [25] to predict the demand for shared bicycles, which can improve the effectiveness and accuracy of a single recurrent neural network (RNN) model. Similarly, Ma et al. [26] proposed a spatial-temporal graph attentional (STGA)-LSTM neural network framework to predict short-term bike-sharing demand using a multi-source data set. Most of these models combine LSTM, improve the prediction accuracy of a single model to some extent, and provide a new method for demand prediction of shared bikes. However, for strongly periodic demand forecasting, there are still inadequate studies in giving full play to the performance of LSTM.

Based on the predicted demand for shared bikes at different locations, optimal bike deployment has been an important subject for operators to balance the demand and supply in space and time. Developing an effective vehicle deployment planning model has been discussed extensively by scholars. For instance, Forma et al. [27] proposed a bike static reset deployment method and designed a three-step heuristic algorithm to make real-time responses for bike demand and vacant piles at sites. Angeloudis et al. [28] redistributed the allocation lines by developing a dynamic bike-sharing allocation algorithm and demonstrated the model's effectiveness through numerical examples. Jost et al. [29] proposed a dynamic pricing strategy to improve the imbalance between the supply and demand of shared bikes based on the idea of self-distribution balance in public bike spaces. However, results showed that although the pricing strategy could improve the imbalance issue, it would reduce travelers' willingness to use shared bikes. Jiménez et al. [30] combined the real-time changes in shared bike demand by optimizing the deployment paths. A station-based hybrid deployment model was proposed to effectively reduce the deployment cost. The optimal allocation path based on the minimum cost has been a major focus in many studies. Furthermore, Raviv et al. [31] focused on the stochastic and dynamic changes in public bike demand to propose an optimization model based on the static bike relocation problem. The model uses a time-dependent objective function to optimize vehicle paths, which can effectively assist path and inventory decisions.

Reviewing the literature on the allocation strategy of shared bikes shows that most of the existing allocation schemes are based on simple manual allocation, and the allocation schemes do not cater to the hotspot usage areas of shared bicycles from a station perspective. In other words, they did not consider the demand for shared bikes from stations at different times, resulting in more significant problems in the management and operation of shared bikes in the surrounding rail transit stations. On the other hand, the existing prediction models have been developed independently, and their contribution to the optimal allocation of shared bikes has not been examined. Given this shortcoming, we propose an optimal shared bike allocation model incorporating the demand heterogeneity of shared bikes. More specifically, we propose a hybrid model to predict the demand for shared bikes by giving full play to the advantages of SARIMA and LSTM models and a deployment model to meet station-wide bike-sharing demand under the premise of cost minimization.

## Methodology

### Bike-Sharing Demand Forecasting Model

The bike-sharing order data has an obvious time pattern, and its generation and ending have clear time points, so the time-by-time order data derived from the statistics belong to the classical time-series data. Classical models in time-series research include ARMA, ARIMA, and SARIMA. These regression-based models can effectively deal with the linear part of time-series data but are not sensitive to the nonlinear characteristics.

In order to overcome the disadvantage of linear models in prediction with nonlinear features, recent studies have increasingly applied machine learning methods that are considered to effectively deal with the nonlinear association in the data, including traditional neural networks, deep learning, models such as recurrent neural network and LSTM, to enhance the model predictability. However, these machine learning methods depend highly on data and model selection. The results obtained also lack a certain degree of interpretability. Thus, recent activities regarding the synergy between theory-driven and data-driven methods have emerged [32].

In this study, we contend that the time-dependencies in the time-series data can be best captured using the time-series data-based models, while the residual (random component) in the linear model may be best captured with a highly nonlinear structure in deep learning. In other words, we propose a model that integrates SARIMA and LSTM models, a SARIMA-LSTM hybrid model, such that the ridership of shared bikes can be predicted using advanced machine learning models while simultaneously maintaining the statistical features of the time-series data. In the following sections, we will first present the prediction models such as the SARIMA and LSTM models and focus on the hybrid version of the two models. Because the prediction model generates input data to optimize bike deployment, we also present the optimization model and explain how the two models are integrated.

#### SARIMA Model

SARIMA is an autoregressive model for seasonal sequences developed based on the ARIMA model. By decomposing the time series into a regular time series and seasonal cycles, the model can eliminate the interference of periodic changes and improve its prediction accuracy. The model is generally denoted as ${\text{SARIMA(}}p,d,q{)(}P,D,Q{)}s$ and the expression is shown in Eq. (1).

1 $$\phi_{p} \left( K \right)\varphi_{P} \left( K \right)(1 - K)^{d} (1 - K^{s} )^{D} Y_{t} = \theta_{q} \left( K \right)\omega_{Q} \left( K \right)\varepsilon_{t}$$

where $Y_{t}$ is the time series; $K$ represents the lag operator, which serves to regress the sequence by one or more cycles; $s$ represents cycles, such as seasonal cycles $s = 4$, monthly cycles $s = 12$, and so on; $\varepsilon_{t}$ is the white noise sequence conforming to normal distribution; $d$ and $D$ represent the number of nonseasonal and seasonal differences, respectively; $\phi_{p} \left( K \right)$ and $\varphi_{P} \left( K \right)$ are functions describing the relationship between adjacent moments of time series $Y_{t}$, as shown in Eqs. (2) and (3), which $p$ and $P$ mean the order of the nonseasonal and seasonal autoregressive terms, respectively.

2 $$\phi_{p} (K) = 1 - \alpha_{1} K - \alpha_{2} K^{2} - \cdots - \alpha_{p} K^{p}$$

3 $$\varphi_{P} (K) = 1 - \beta_{1} K^{s} - \beta_{2} K^{2s} - \cdots - \beta_{P} K^{Ps}$$

where $\theta_{q} \left( K \right)$ and $\omega_{Q} \left( K \right)$ are relational functions describing the seasonal period of time series $Y_{t}$. The expressions are shown in Eqs. (4) and (5), where $q$ and $Q$ represent the nonseasonal and seasonal moving average term orders, respectively.

4 $$\theta_{q} (K) = 1 + \chi_{1} K + \chi_{2} K^{2} + \cdots + \chi_{q} K^{q}$$

5 $$\omega_{Q} (K) = 1 + \lambda_{1} K + \lambda_{2} K^{2s} + \cdots + \lambda_{Q} K^{Qs}$$

**Fig. 1:**
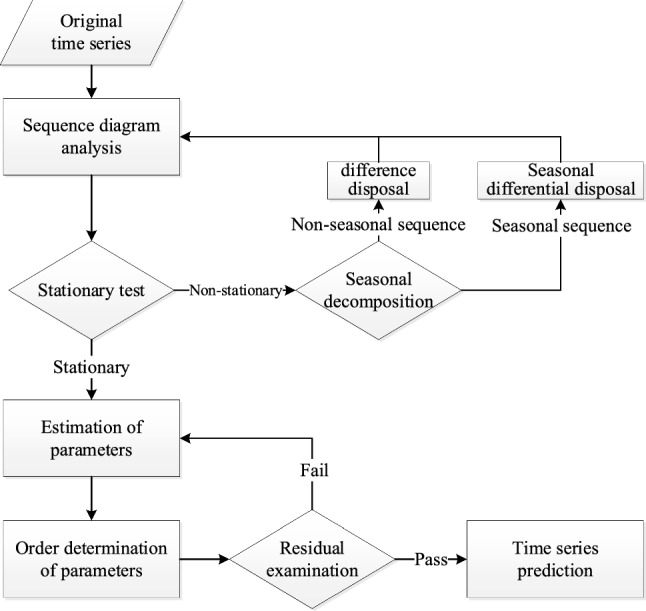
The implementation process of the SARIMA model

The process of implementing a SARIMA model is depicted in Fig. 1. Firstly, the time-series data are analyzed by time-series plot to determine the nonstationary series. Then the nonstationary series is differenced and seasonally differenced to obtain the minimum values of *d* and *D*. Next, the model’ parameters are estimated and determined, and the residuals of the model are tested. Finally, numerical predictions are made by the eligible ${\text{SARIMA(}}p,d,q{)(}P,D,Q{)}s$.

#### LSTM Model

**Fig. 2:**
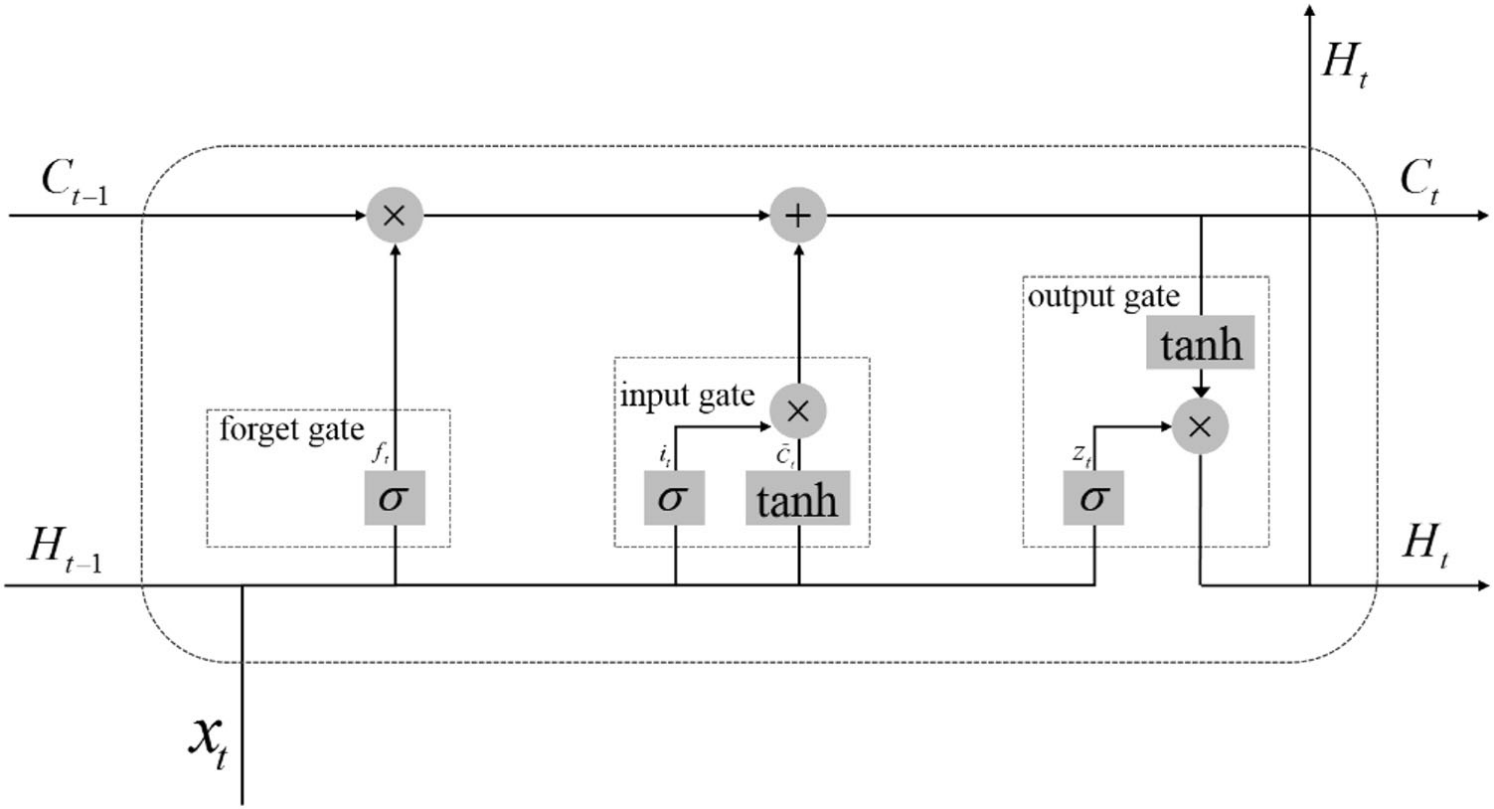
LSTM standard cell structure diagram

LSTM is used to solve the long-term dependency and gradient problems, and its basic unit is shown in Fig. 2.

According to Fig. 2, LSTM mainly consists of three gates: input gate, forget gate and output gate. The process of LSTM is to pass the input sequence $\{ x_{1} ,x_{2} ,...,x_{t} \}$ through an encoder-decoder and finally output the predicted sequence $\{ y_{1} ,y_{2} ,...,y_{t} \}$.$x_{t}$, $C_{t}$ and $H_{t}$ means the input value, the regenerative cell state value and the output cell hidden state value at time $t$, respectively.

First, the forget gate is passed, and the forget gate operation expression is shown in Eq. (6), where $H_{t - 1}$ is the hidden state value of the cell at the previous moment and $x_{t}$ is the input value at this moment, and $\sigma$ function is used to output a weight $f_{t}$ between 0 and 1, which is taken as the probability value of the node state at the time before the neural unit forgets.

6 $$f_{t} = \sigma \left( {W_{f} \left[ {H_{t - 1} ,x_{t} } \right] + b_{f} } \right)$$

In the input gate, the state information of the candidate cell $\tilde{C}_{t}$ can be generated from the $\tanh$ tanh function, and $\sigma$ function generates a weight $i_{t}$ to determine the size of the state information of the candidate cell.

7 $$\tilde{C}_{t} = \tanh (W_{c} \left[ {H_{t - 1} ,x_{t} } \right] + b_{c} )$$

8 $$i_{t} = \sigma \left( {W_{i} \left[ {H_{t - 1} ,x_{t} } \right] + b_{i} } \right)$$

Combining with the cell state at the previous moment $C_{t - 1}$, the regenerative cell state at this moment $C_{t}$ is shown in Eq. (9).

9 $$C_{t} = f_{t} \cdot C_{t - 1} + i_{t} \cdot \tilde{C}_{t}$$

Finally, the value of the output gate $z_{t}$ can be calculated from Eq. (10), and the current hidden state $H_{t}$ can be calculated by combining the current state $C_{t}$ with Eq. (11). By processing $H_{t}$ through the decoder, the predicted sequence $\{ y_{1} ,y_{2} ,...,y_{t} \}$ is obtained.

10 $$z_{t} = \sigma \left( {W_{z} \left[ {H_{t - 1} ,x_{t} } \right] + b_{z} } \right)$$

11 $$H_{t} = z_{t} \cdot \tanh (C_{t} )$$

In the above equations, *W*_*f*_*, W*_*c*_*, W*_*i*_*, W*_*z*_ are the weight matrixes, *b*_*f*_*, b*_*c*_*, b*_*i*_*, b*_*z*_ are bias vectors. These are exactly the eight sets of parameters that machine learning is designed to learn to obtain better predictions.

#### SARIMA-LSTM Hybrid Model

The SARIMA-LSTM hybrid model combines the time-series prediction model and the neural network prediction model to predict the time-series volume of shared bikes,$\left\{ {Y_{t} } \right\}\left( {t = 1,2,3,...,n} \right)$ . According to the characteristics of different models, $\left\{ {Y_{t} } \right\}$ can be divided into linear structure $\left\{ {L_{t} } \right\}$ and nonlinear structure $\left\{ {e_{t} } \right\}$, which are expressed in Eq. (12).

12 $$Y_{t} = L_{t} + e_{t}$$

**Fig. 3:**
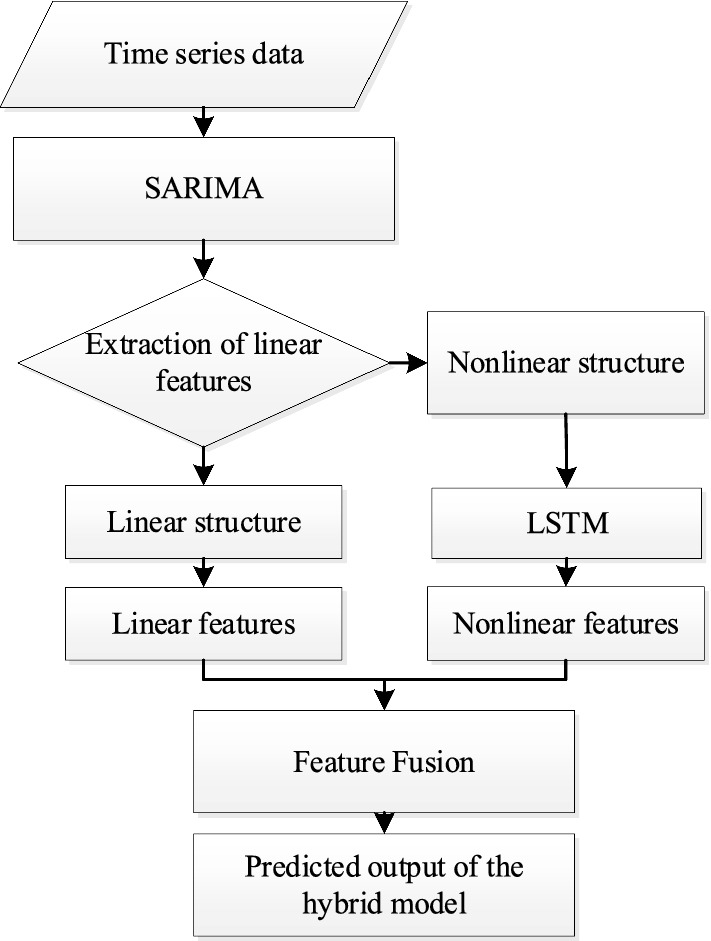
Structure of hybrid prediction model.

Because the hybrid model involves parameter estimation of SARIMA and the training of LSTM, a joint model estimation process is needed. The structure diagram of the hybrid model is shown in Fig. 3, and the three core steps are described below.

1. Extract linear features of the data.
  The data set was divided into a training set and a test set, which were built with a SARIMA model using SPSS. Then the linear fitting value $\hat{L}_{t}$ of shared bike time-series data and linear value $\hat{L}_{t + 1} ,\hat{L}_{{t{ + 2}}} ,...,\hat{L}_{{t{\text{ + n}}}}$ of future period $n$ are obtained by the SARIMA prediction.

(2) Analyze the nonlinear characteristics of data.

After SARIMA modeling and prediction, the residual sequence reflecting the nonlinear structure of the data can be obtained, as shown in Eq. (13).

13 $$e_{t} = Y_{t} - \hat{L}_{t}$$

Then, the Keras deep learning library in Python and the TensorFlow package are used to build the LSTM network model. The residual sequence is used as the input value for training. After the parameters are stabilized, the model is used to predict the nonlinear value $\hat{e}_{t + 1} ,\hat{e}_{t + 1} ,...,\hat{e}_{t + n}$ in future period $n$.

(3) The predicted value of future period $n$ is obtained by using the hybrid model prediction.

14 $$\hat{Y}_{t + i} = \hat{L}_{t + i} + \hat{e}_{t + i} \left( {i = 1,2,...,n} \right)$$

#### Evaluation Metrics

To assess the predictive performance of the models, mean absolute error (MAE), root mean square error (RMSE), and mean absolute percentage error (MAPE) are adopted. MAE and RMSE reflect the absolute error in the predicted and actual values, while MAPE reflects the relative percentage error. Smaller values of MAE and RMSE indicate a smaller fitting deviation of the model, thus better accuracy in prediction. In MAPE cases, a value lower than 10% has been considered empirically to be satisfactory. The formulas of the three indicators are shown below:

15 $$MAE = \frac{1}{n}\mathop \sum \nolimits_{t = 1}^{n} \left| {\hat{y}_{t} - y_{t} } \right|$$

16 $$RMSE = \sqrt {\frac{1}{n}\mathop \sum \nolimits_{t = 1}^{n} \left( {\hat{y}_{t} - y_{t} } \right)^{2} }$$

17 $$MAPE = \frac{1}{n}\mathop \sum \nolimits_{t = 1}^{n} \left| {\frac{{\hat{y}_{t} - y_{t} }}{{y_{t} }} \times 100} \right|$$

where $\hat{y}_{t}$ represents the predicted value of the model, $y_{t}$ represents the actual value, and $n$ represents the number of samples.

### Bike-Sharing Allocation Strategy

By physically moving the bikes from one location to another, the demand in target areas can be matched, while the over-allocation in original areas can be released. Before redeployment begins, a redeployment plan will be developed to achieve a better balance. The plan usually includes transportation volumes and routes, with transportation costs being the essential basis for its development.

#### Bike-Sharing Allocation Process

The deployment of bike sharing is a system project. Preparing a deployment plan based on order data also requires the full cooperation of deployment command points, deployment locations, and deployment experts. In order to standardize the operational process of deployment, we summarize the management methods of different companies deploying shared bikes and propose the following deployment strategies.

1. After the morning peak, the enterprise allocation center calculates the inflow and outflow of shared bikes at various rail transit stations and deployment sites based on the order data uploaded to the system.
2. According to the combined prediction model, input the inflow and outflow of shared bikes in the morning peak hours, predict the inflow and outflow of shared bikes in the peak hours and evening peak hours of all stations, and determine the export and import volume of the target area around the urban rail transit station and the upper limit of the number of shared bikes that can be accommodated and supplemented by the allocation station.
3. Aiming at the company's minimum deployment cost, establish a shared bike deployment model based on the transportation problem of production and sales balance, and solve a specific shared bike allocation plan.

The allocation center sends the allocation plan to the specialists around each site. The allocation specialists complete the allocation of shared bikes in the allocation skylight according to the allocation instructions and report them in time.

#### Deployment Areas

Analysis of the allocation strategy shows that the division of bike-sharing allocation areas is the basis for allocating work. In other words, we need to know which station needs to move bikes out and which one needs to replenish before we can optimize.

As there are no fixed stations for bike sharing, users can rely on intelligent location devices such as onboard GPS to park and pick up their bikes anytime. That poses a challenge for deployment. The shared bike drop-off area is divided into different deployment units to simplify the deployment of shared bikes. In the actual scheduling process, the deployment center often determines the areas to be assigned based on historical experience, big data, analysis and dispatcher's instructions—in other words, without long transport distances for cost savings in the deployment. According to the analysis of shared bike data, 77.4% of shared bikes are manually assigned a straight-line distance of 3 km or less. Therefore, the straight-line distance between stations within the setup dispatch unit does not exceed 3 km.

Based on the deployment units, the deployment sites of shared bicycles can be further divided. The areas around the urban rail transit station were considered, in general, a hot spot for the use of shared bikes; another study showed that shared bikes going to or leaving a station were distributed within 2 km of the station [33].

To disperse or supplement shared bicycles around stations, it is necessary to set up additional deployment sites. We filtered the latitude and longitude at the starting and ending points of bike-sharing orders within 2 km of all stations. We used a *k*-means clustering algorithm to identify hot spots of bike-sharing usage. A 500-meter radius of the cluster center was used as the deployment site for the shared bikes.

#### Amount of Demand for Deployment

**Fig. 4:**
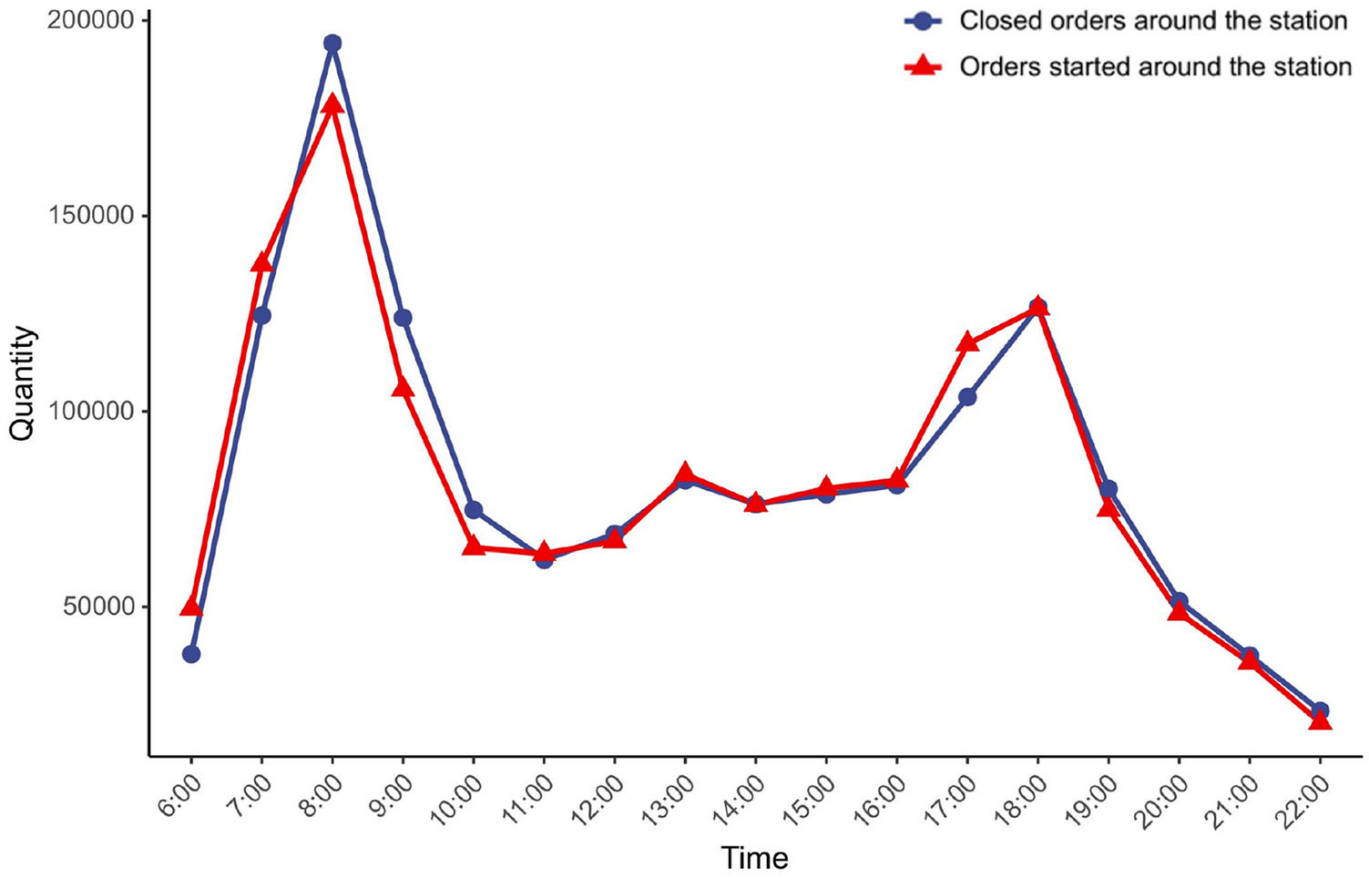
Usage of shared bicycles around urban rail transit stations

Here, we analyzed the number of shared bikes used around stations (Fig. 4).

Figure 4 shows that there are two distinct peaks in the volume of bike-sharing usage around rail stations. After calculation, the number of bicycles used during the peak period accounts for 40% of the whole day. Combined with the actual operation strategies of the operating companies, dynamic deployment with a high degree of refinement would result in excessive operating costs. Therefore, this paper still adopts the deployment scheme based on the skylight time and determines the skylight time as from 9:00 to 17:00.

The allocation demand in each area is calculated according to the flow difference, which refers to the difference between the inflow and outflow of regional shared bikes in the same period. The deployment requirement for each place can be calculated by Eq. (18).

18 $$\Delta D_{k} = \sum\limits_{t} {({\text{Flow\_in}}_{t} - {\text{Flow\_out}}_{t} } )$$

The inflow and outflow of shared bikes are predicted according to the SARIMA-LSTM combination model. Then, we calculated the allocation demand in the stations and the allocation site areas, respectively. For the urban rail transit stations, when $\Delta D_{k} > 0$, the shared bikes flow into this station, and the shared bikes need to be transferred out to the surrounding allocation stations, which is called the export urban rail station; when $\Delta D_{k} < 0$, the shared bikes flow out of this station, and the shared bikes need to be transferred into this station from the surrounding allocation stations, which is called the import urban rail station.

For the deployment sites, when $\Delta D_{k} > 0$, the continuous inflow of shared bikes in this station can provide a certain number of shared bikes for outflow urban rail transit stations with insufficient shared bikes nearby, which are supplementary deployment sites; when $\Delta D_{k} < 0$, the shared bikes in this site continue to flow out, so there is a certain capacity for shared bikes, which can accommodate the excess shared bikes of nearby inflow urban rail transit stations, which are the accommodation deployment sites.

#### Bike-Sharing Allocation Model

By physically moving the bikes from one location to another, the demand in target areas can be matched while the over-allocation in original areas can be released. Before redeployment begins, a redeployment plan will be developed to achieve a better balance. The plan usually includes transportation volumes and routes, with transportation costs being the essential basis for its development. The allocation task is to allocate the shared bikes to be transferred from all export urban rail stations to import urban rail stations and accommodation deployment sites. At the same time, the shared bikes required by all import urban rail stations can be transferred in from export urban rail stations and supplementary deployment sites. The critical point is that we need to minimize the costs incurred in shipping. Therefore, based on the balanced transportation model, we allocate shared bikes with the minimized cost as the objective function, as shown in Eq. (19).

19 $$\begin{aligned} & \min \left( {\sum\limits_{i = 1}^{m} {\sum\limits_{j = 1}^{n} {\gamma_{ij} + } } \sum\limits_{i = 1}^{m} {\sum\limits_{j = 1}^{N} {\gamma_{ij} } } + \sum\limits_{i = 1}^{M} {\sum\limits_{j = 1}^{n} {\gamma_{ij} } } } \right) \\ & \quad s.t.\left\{ \begin{gathered} \hfill \sum\limits_{j = 1}^{n} {x_{ij} = a_{i} ,i = 1,2,3,...,m \, \quad \, (1)} \\ \hfill \sum\limits_{j = 1}^{n} {x_{ij} \le p_{i} ,i = 1,2,3,...,M \, \quad \, (2)} \\ \hfill \sum\limits_{i = 1}^{m} {x_{ij} = b_{j} ,j = 1,2,3,...,n \, \quad \, (3)} \\ \hfill \sum\limits_{i = 1}^{m} {x_{ij} \le q_{j} ,j = 1,2,3,...,N \, \quad \, (4)} \\ \hfill x_{ij} = 0,i = 1,2,3,...,M;j = 1,2,3,...,N \, \quad \, (5) \\ \hfill x_{ij} \ge 0,i,j{\text{ are not all deployment stations}}\quad \, (6) \\ \end{gathered} \right. \\ \end{aligned}$$

The definitions of the parameters in the formula are shown in Table 1.

**Table 1:** Model parameters and definitions

Parameters	Definition
$\gamma_{ij}$	The deployment cost from station $i$ to $j$
$x_{ij}$	the volume of bikes transported from *i* to *j*, and $x_{ij}$ is an integer
$m$	The number of export urban rail station
$M$	The number of supplementary deployment sites
$n$	The number of import urban rail station
$N$	The number of accommodation deployment sites
$a_{i}$	The number of bikes that need to be called out for the *i*th exit urban rail station
$b_{i}$	Demand quantity for bicycles of the *j*th import urban rail station
$p_{i}$	The number of bikes that need to be called out for the *i*th supplementary deployment sites
$q_{i}$	Demand quantity for bicycles of the *j*th accommodation deployment sites

The effect of constraints ① and ③ is to transport the excess bikes to the stations that need resupply. Constraint conditions ② and ④ mean that the number of shared bikes allocated at the supplementary and accommodation allocation stations should not exceed its upper limit. The last two constraints mean that the allocation of shared bikes cannot occur between the allocation stations.

The costs incurred for deployment in this paper consist of labor and transportation costs, which are calculated in Eqs. (20, 21, 22 and 23). The definitions of the parameters in the formula are shown in Table 2.

20 $$\gamma_{ij} = \gamma_{H,ij} + \gamma_{T,ij}$$

21 $$\gamma_{H,ij} = x_{ij} \cdot L_{ij} \cdot v$$

22 $$v = \frac{{\text{monthly salary}}}{{{\text{distance}} \cdot {\text{number of bikes}}}}$$

23 $$\gamma_{T,ij} = 2\frac{{x_{ij} }}{n}s \cdot L_{ij}$$

**Table 2:** Parameters and definitions of deployment costs

Parameters	Definition
$\gamma_{H,ij}$	The allocated labor cost, which is calculated based on interviews with staff
$L_{ij}$	The shortest road network distance from location $i$ to location $j$, which is obtained by Baidu Maps API
$v$	The labor cost per km of shared bikes allocation, in this paper $v = 1$ according to our interview results
$\gamma_{T,ij}$	The allocated transportation cost, which is mainly influenced by the fuel consumption and maintenance cost of the transport vehicle
$n$	The maximum number of shared bikes that can be accommodated by each transport vehicle, in this paper, $n = 60$
$s$	The driving cost per kilometer of the transport vehicle, we supposed $s = 2{\text{yuan/km}}$ according to our interview results

The model can be solved by the table on the operating method. We implemented such an optimization algorithm via MATLAB, in which the initial feasible solution uses the Vogel method, and the optimal solution is determined by closed-loop adjustment.

## Empirical Results

In this section, we introduce the empirical analysis conducted based on the data in Beijing, the capital of China. First, we describe the data and fields being analyzed. Then the effectiveness of the hybrid model is verified based on the data. Taking the Xicheng District of Beijing as the basic dispatching unit, the allocation process for shared bikes around urban rail transit stations within the dispatching unit is elaborated in detail, and the deployment scheme for shared bikes around urban rail transit stations in the Xicheng District is formulated to verify the applicability of the proposed model.

### Data and Research Area

The Xicheng District is located in the west of the central city area. Some stations have a higher number of shared bikes than other areas during morning and evening peak hours, which is more likely to have the imbalance problem between supply and demand of shared bikes. The *k*-means was used to filter the possible deployment sites in the Xicheng District. According to the silhouette coefficient and within-groups sum-of-squares errors (SSE), the clustering results show the best performance when *k* = 932. The results show that there are 22 known urban rail transit stations and 28 qualified allocation sites were selected for deployment.

The primary data set used in this study is the payment transaction records of Mobike in Beijing from April to June 2018. Mobike is one of the first bike-sharing enterprises to enter the Chinese market and has recorded a huge number of rental trips over the years. Through vehicle smart locks and GPS positioning devices embedded in the bikes, location data are recorded as well as the registration data of users, including user ID, bike ID, and latitude and longitude information of start and end locations. Mobike generates about 900,000 orders daily, and some of the order data contain abnormal data due to various disturbing situations, such as smart lock failure, unstable GPS signal, and human error. These data will interfere with the accuracy of model results. Thus, before the analysis, a data cleaning process is implemented to screen out standard order data. Abnormal data in this study are identified based on the following five criteria:

1. Records having empty values in crucial fields such as order time, latitude, and longitude
2. Abnormal data repeatedly uploaded to the system in the same order
3. Abnormal data where the end time is earlier than the start time and multiple bikes are parked at the same location
4. Data on starting or ending locations (longitude and latitude) outside the administrative geographical scope of Beijing
5. Abnormal data with usage time less than 2 minutes or longer than 3 hours and driving distance less than 150 m or more than 10 km

In addition to Mobike data, we use the data for Beijing rail transit stations, road networks, and administrative areas. These data are downloaded from OpenStreetMap. We use ArcGIS software to calculate the inflow and outflow of shared bikes around the Xicheng District. Moreover, we also call the Baidu Maps API to generate the shortest path data, which is used for the cost calculation of the optimization model.

### Prediction Results of the Hybrid Model

To verify the effectiveness of the hybrid model, we take the surroundings of Fuchengmen station as an example and use SARIMA, LSTM, and SARIMA-LSTM models for prediction. There were 35 valid data sets on orders for shared bikes around the Fuchengmen subway station. The time interval for the analysis of each data set was from 6:00 to 21:00 with a granularity of 1 hour. In this paper, the first 34 sets of data were used as the training set for the prediction model, and the last set of data was predicted.

The training set data were first analyzed using the SARIMA model. Based on the results of the data processed by SARIMA modeling, when the model performance was optimal and the error was minimal, the parameter of ${\text{SARIMA}}(p,d,q)\;(P,D,Q)s = {\text{SARIMA}}\;(1,0,2)\;(0,1,1)\;16$ was used for prediction.

An LSTM model was then used to perform the same prediction work. The LSTM network model framework in this paper was built using the Keras deep learning library in Python and the TensorFlow package. When using the LSTM model, a set of hyperparameters (as listed in Table 1) needs to be determined because different settings affect the prediction results. To avoid difficult convergence of the model or excessive computational work, the number of hidden layers ${\text{lstm\_size}}$ was set to 1. because of the small amount of data, ${\text{time\_step}} = 15$,${\text{ batch\_size}}\;{ = }\;{2}$, and ${\text{epochs}}\;{ = }\;{100}$ were set to ensure accuracy. MAE was used as the loss, which can better characterize the distribution of standard data. The model used Adaptive Moment Estimation (Adam) as the optimizer during the training process because it has a tremendous advantage over the optimizer in terms of learning speed and convergence speed (Table 3).

**Table 3:** The main parameters in the LSTM model

Name of the parameter	Meaning
Lstm_size	Number of hidden layers
Time_step	Number of blocks of the expanded LSTM
Batch_size	Number of rows in each batch feeding into the LSTM
Epochs	Number of iterations to train the model
Optimizers	Method for adjusting the weight of each node
Loss	Method for calculating the difference between the output of a neural network and the sample markers

**Fig. 5:**
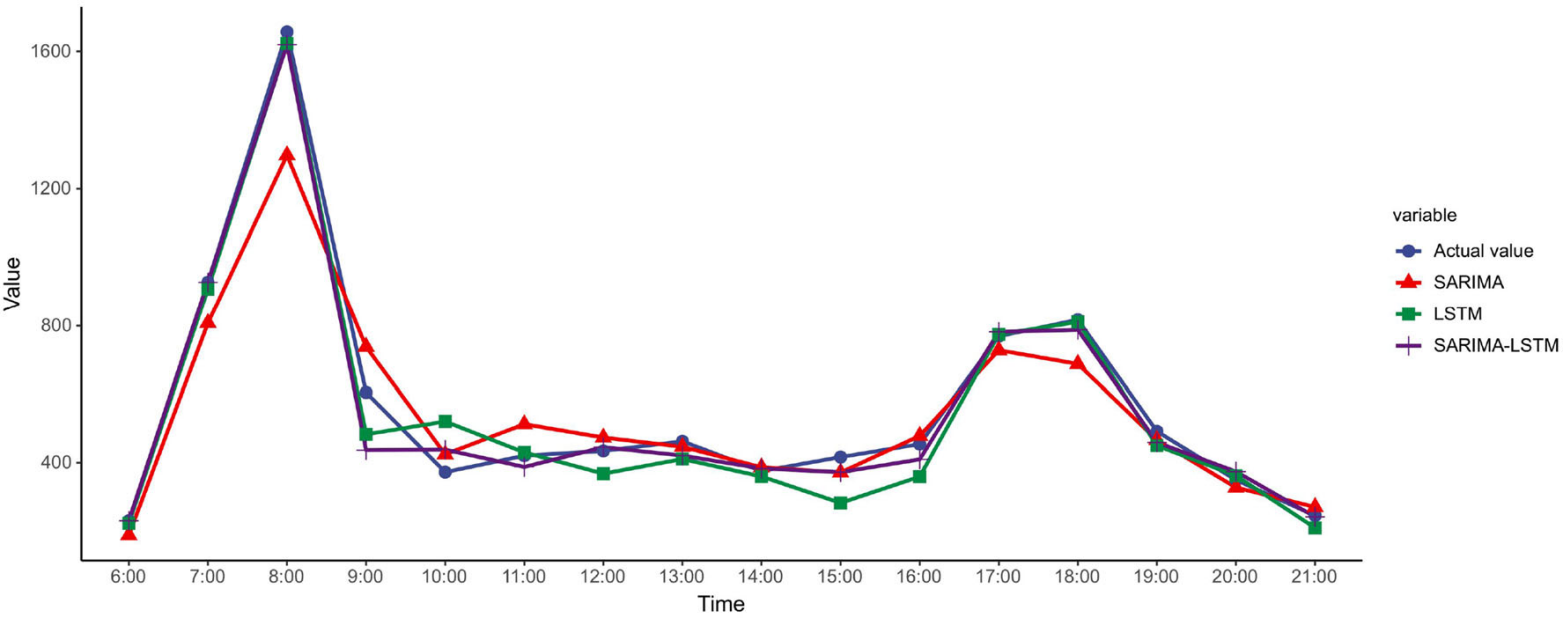
Comparison of prediction results of different models

Finally, we ran the SARIMA-LSTM hybrid model with the same parameters as the separate predictions. The prediction results of the three models are summarized in Fig. 5, and the effect evaluation is shown in Table 4. The ‘original' in Fig. 5 meant the actual sequence data of this day. It can be seen from Fig. 5 that the SARIMA-LSTM model, which is a combination of two different models, not only accurately captured the linear features in the data but also better grasped the remaining trend of the data and had a high fit with the actual data. The hybrid model was able to maximize the closeness to the actual data for most of the time, especially during the peak hours, and it could also be observed that the LSTM also performed relatively well. In other periods, the LSTM showed significant deviations from the actual results compared to the other models. In contrast, the hybrid model effectively took advantage of the two separate models, and the predictions did not deviate much.

**Table 4:** Effect evaluation of different prediction models

Models	Evaluation indexes		
	MAE	RMSE	MAPE (%)
SARIMA	63.085	101.136	22.486
LSTM	58.480	99.772	10.642
SARIMA-LSTM	43.513	54.467	9.765

Table 4 shows that the hybrid model had smaller MAE, RMSE, and MAPE than the other models. It is worth mentioning that the MAPE of the hybrid model was less than 10%. The results demonstrated that the model with a hybrid of SARIMA and LSTM is more accurate than when used separately in predicting bike-sharing time-series data.

### Bike-Sharing Allocation

**Fig. 6:**
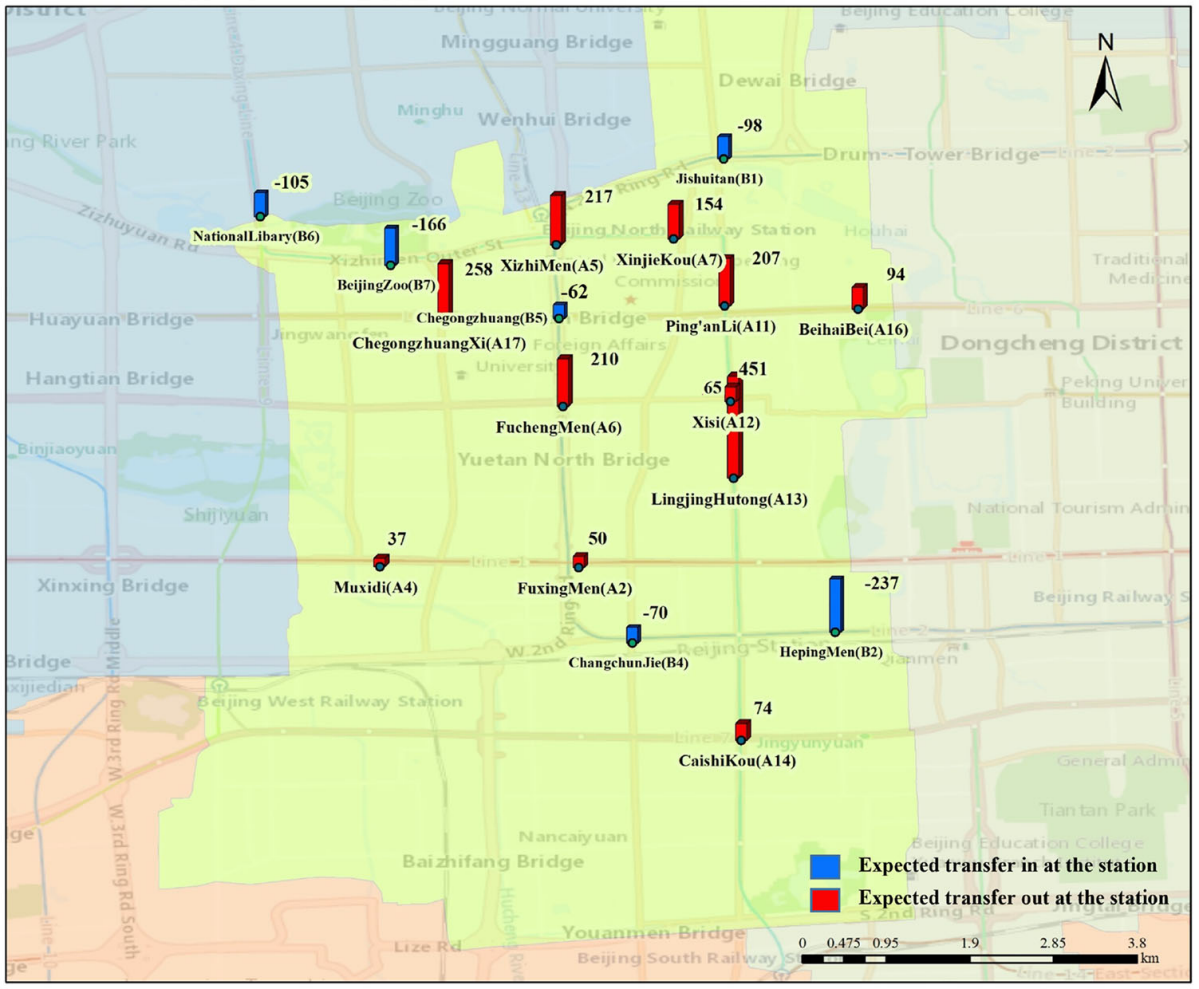
Participating deployment sites of urban rail transit stations and deployment volume

**Fig. 7:**
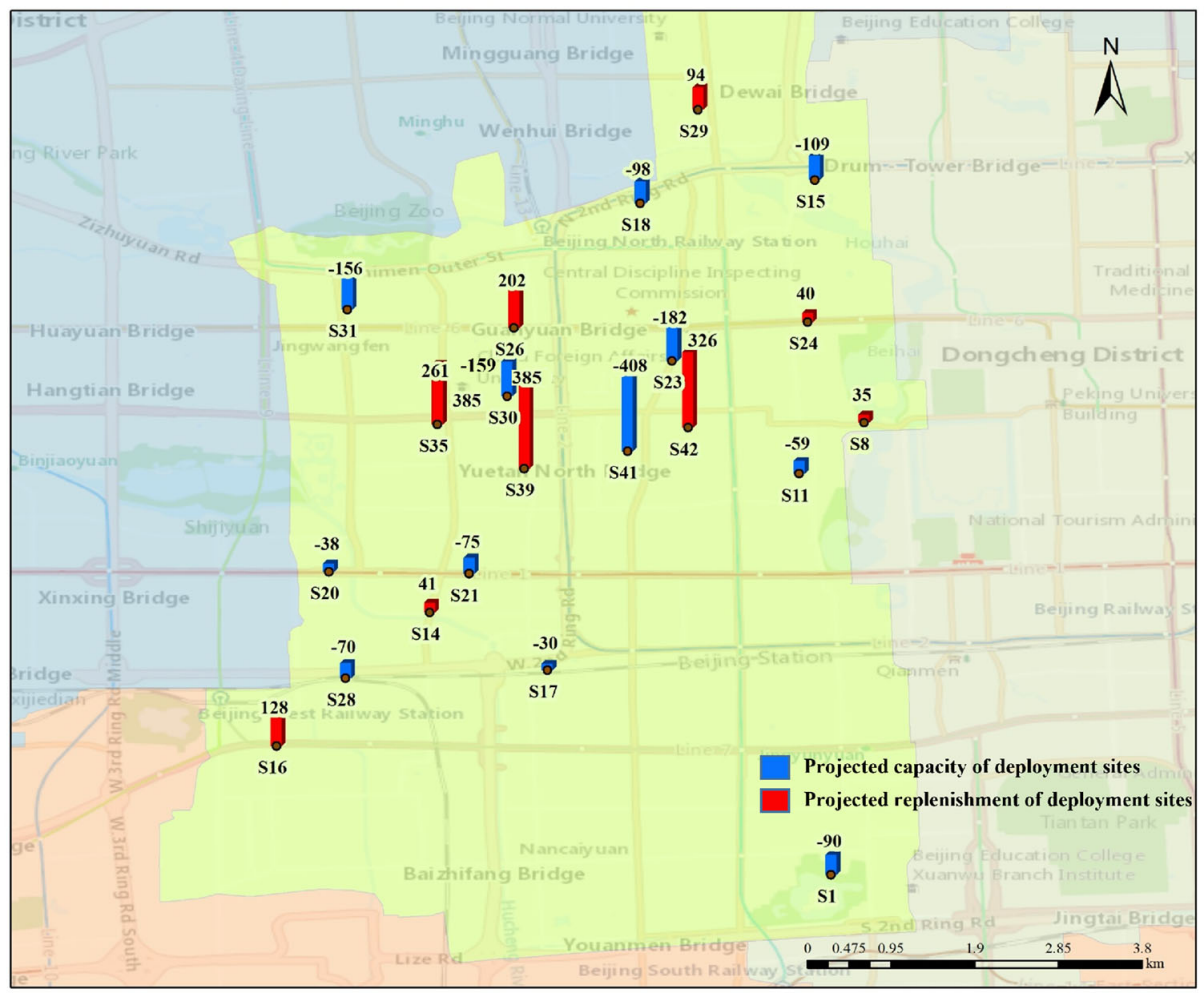
Participating deployment sites and deployment volume

With the forecast data, an effective and dynamic deployment plan can be developed in advance to meet the demand of residents for bike-sharing usage. Because of the low demand for shared bikes at some sites, when the demand at a site is less than half the maximum capacity of a dispatched bike, i.e., 30 bikes, it is considered that the site will not participate in the deployment. After the statistical screening, the Xicheng District participated in the deployment of 17 urban rail transit stations, including 11 exit stations with 1817 bikes and six import stations with a total of 738 bikes. These stations and the demand for shared bikes are plotted using GIS, as shown in Fig. 6. There are 21 participating deployment sites, including nine supplemental sites for up to 1512 supplemental bikes and 12 accommodating sites for up to 1474 bikes. We show these sites in Fig. 7.

**Fig. 8:**
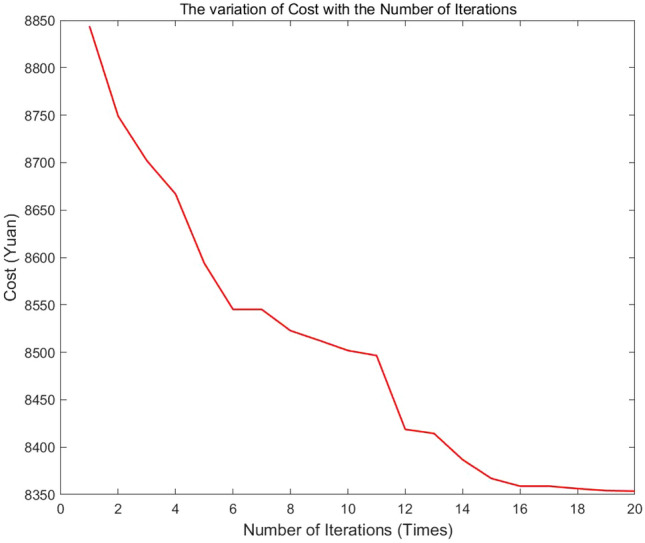
The variation in cost with the number of iterations

Then the planning was done according to the bike-sharing allocation model. With a total of 3329 bikes to be shipped out and total demand of 2212 bikes, it is necessary to add a virtual allocation center as the 13th accommodation allocation station to accommodate an extra 1117 bikes. Based on the above consideration, the unknown quantities in Eq. (18) were determined where $m = 11,N = 12,M = 9,n = 6$. The optimal deployment scheme was obtained after 20 iterations of running the algorithm in MATLAB. The iterative process is shown in Fig. 8, and using this option yields a minimum total cost of 8353.6 yuan.

**Fig. 9:**
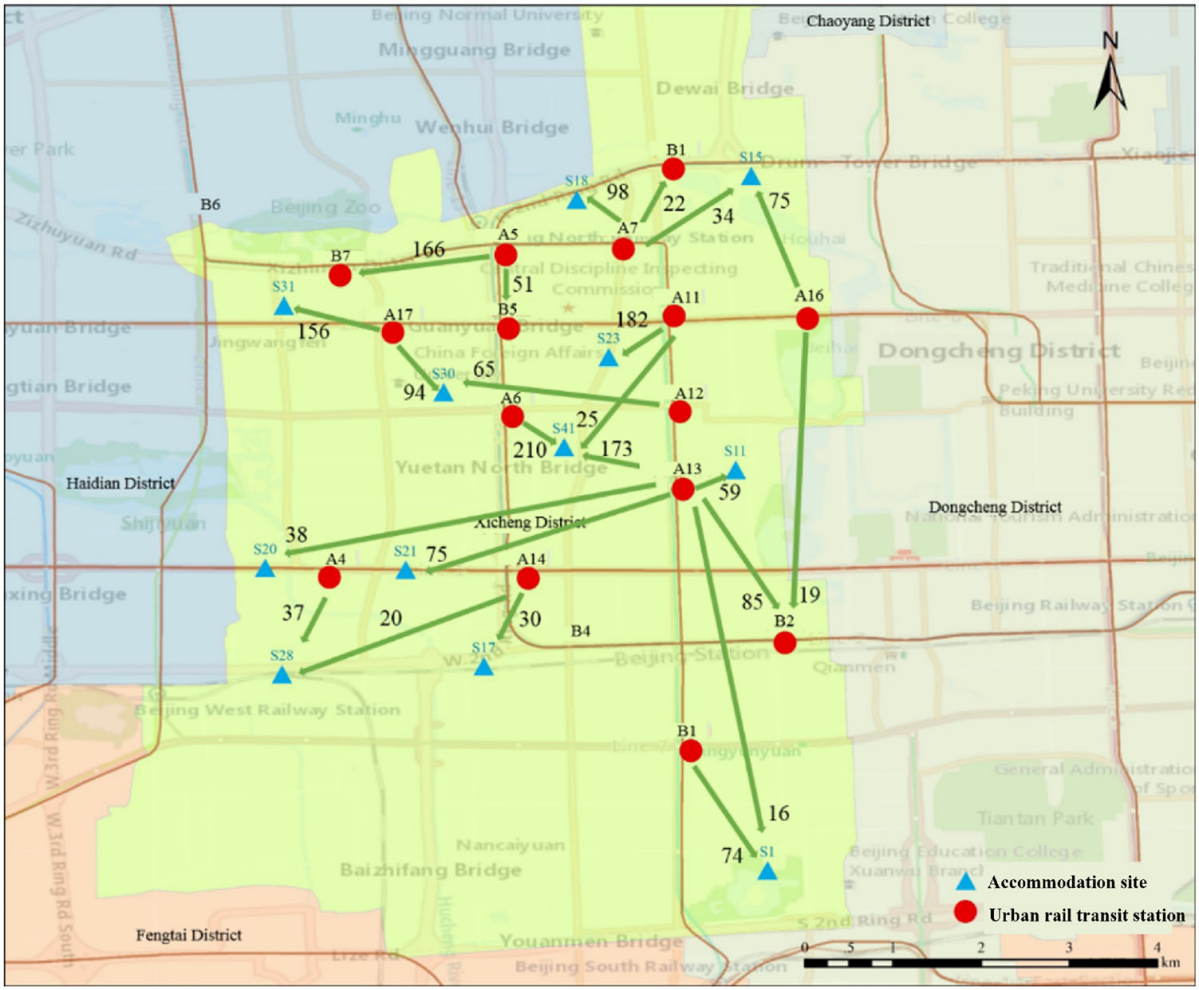
Deployment schematic diagram of export stations

**Fig. 10:**
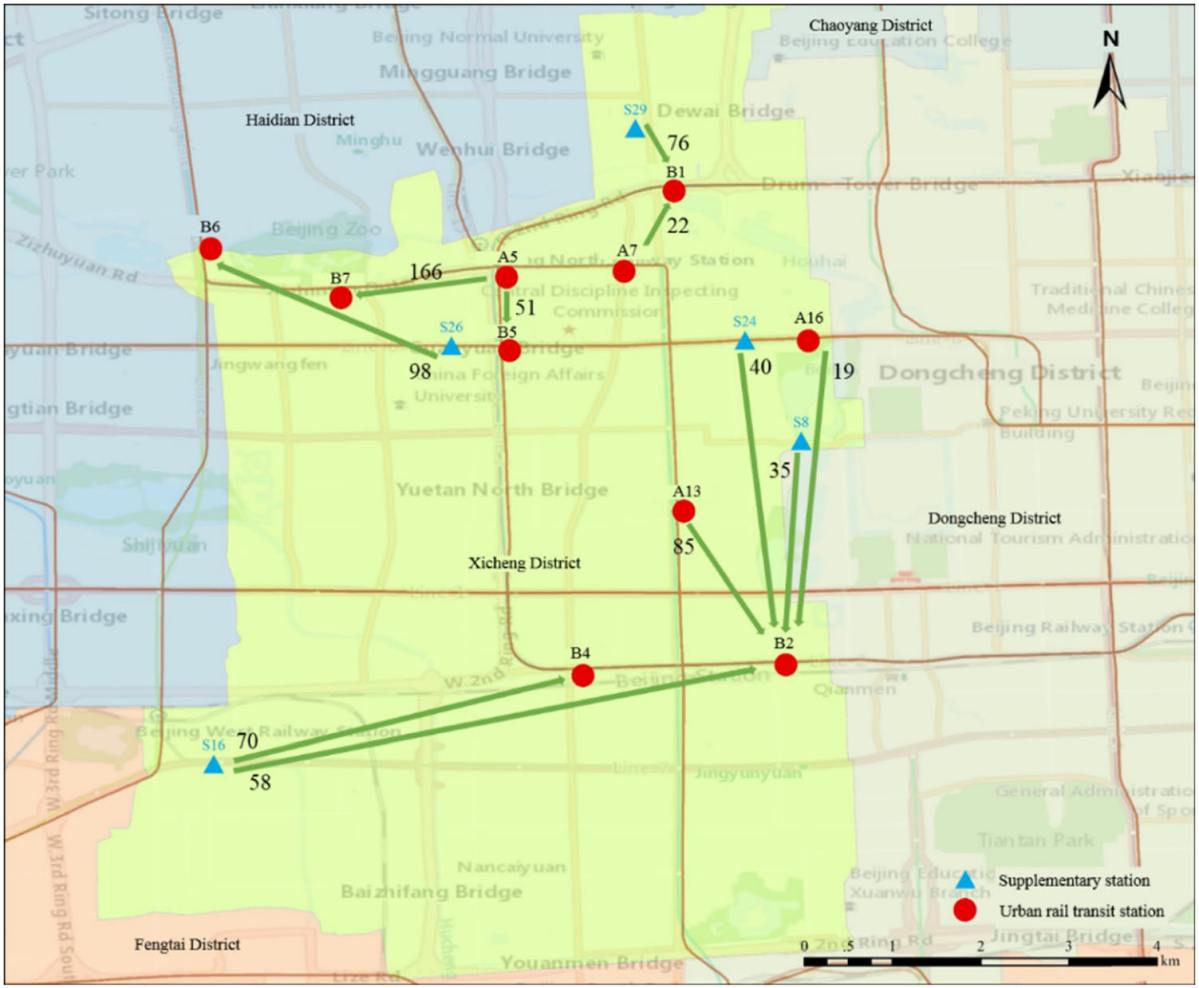
Deployment schematic diagram of import stations

A more visible representation of the deployment plan is shown in Figs. 9 and 10. In particular, Fig. 9 shows which stations need to transport the excess bikes to deployment sites. Figure 10 shows which stations need to replenish bikes from nearby deployment sites. The amount of transportation was marked. Finally, the allocation process was executed according to the method proposed in Sect. 3.2.

Furthermore, a sensitivity analysis is conducted for the labor cost. The result indicates that the current solution is still optimal when $v \in \left[ {0.8, 1.0} \right]$. This result can provide a meaningful reference for managers in pricing and programming.

## Conclusion

As a sustainable transportation mode with the apparent features of green, flexible, and easy access, bike sharing can be used to connect urban rail transit, which contributes to solving not only the first and last mile problems but also transportation decarbonization as a whole. However, the imbalance between the supply and demand of shared bikes in space and time has remained prominent in many cities, resulting in various social problems. For both planners and the convenience of users, an efficient vehicle deployment strategy based on accurate demand prediction is seen as essential to ensure the sustainable benefits of shared bikes in urban transportation systems.

In line with this concern, in this paper, we proposed a hybrid model which focuses on the dynamic deployment of bike sharing around urban rail transit stations based on a machine learning-enabled time-series prediction model. The purpose is to achieve the best deployment plan which minimizes the total cost by balancing the supply and predicted demand at different stations. The proposed hybrid model incorporates the advantages of both SARIMA and LSTM to predict the use of shared bikes.

Using the actual data for the Xicheng District of Beijing, we compared the predicted values of a single model and the hybrid model. Results show that the hybrid model has higher prediction efficiency. This enriches the method for demand prediction of shared bikes. Apart from that, the bike-sharing deployment model around urban rail transit stations was developed to minimize transportation costs based on forecasting data. The resulting bike deployment plan would advise on the bike-sharing imbalance around urban rail transit stations.

This study was conducted by setting the hot spot within 500 m of a station. However, as a flexible transportation mode, the bike-sharing system has many types of hot spots in the city. Therefore, the hot areas for using different types of shared bikes may also be combined to conduct further research on different travel structures and the global deployment optimization of shared bikes. In addition, as this paper focuses on meeting the demand for peak bike-sharing usage, the temporal granularity of the prediction and the establishment of the allocation model were simplified based on the actual deployment of the operating companies. Further research can be conducted to cover dynamic requirements on a time-by-time basis.
